# Poster Session I – Poster of Distinction - A88 MULTI-OMIC FECAL BIOMARKERS PREDICT PERSISTENT METABOLIC DYSFUNCTION–ASSOCIATED STEATOTIC LIVER DISEASE AFTER BARIATRIC SURGERY IN PATIENTS WITH MORBID OBESITY: A PRECISION MEDICINE APPROACH

**DOI:** 10.1093/jcag/gwaf042.088

**Published:** 2026-02-13

**Authors:** P Massara, K J Schwenger, A Taibi, Y Ghorbani, J Pan, S Fisher, T Jackson, A Okrainec, J P Allard, E M Comelli

**Affiliations:** Nutritional Sciences, University of Toronto, Toronto, ON, Canada; Nutritional Sciences, University of Toronto, Toronto, ON, Canada; Nutritional Sciences, University of Toronto, Toronto, ON, Canada; University Health Network, Toronto, ON, Canada; Nutritional Sciences, University of Toronto, Toronto, ON, Canada; University Health Network, Toronto, ON, Canada; University Health Network, Toronto, ON, Canada; University Health Network, Toronto, ON, Canada; University Health Network, Toronto, ON, Canada; Nutritional Sciences, University of Toronto, Toronto, ON, Canada

## Abstract

**Background:**

Metabolic dysfunction-associated steatotic liver disease (MASLD) affects >70% of patients with morbid obesity (MO; body mass index (BMI) ≥ 40 kg/m^2^). Roux-en-Y Gastric Bypass (RYGB) is considered gold standard for treating MO and MASLD, however, there is variability in the response magnitude, and in some patients MASLD persists postoperatively (perMASLD). This variability necessitates predictive methods that can guide personalized treatment strategies before RYGB. We found that the fecal microbiome and miRNome are linked to MASLD pathogenesis and can be promising, non-invasive and reproducible biomarkers to assess MASLD risk. However, they have not been investigated in the context of perMASLD.

**Aims:**

To identify fecal multi-omic biomarkers that predict perMASLD following RYGB in patients with MO.

**Methods:**

We collected liver biopsy samples, stool, serum metabolites, and clinical variables from 61 patients with MO at baseline and again 12 months after RYGB. PerMASLD was defined as MASLD diagnosis at both baseline and 12 months after RYGB. Fecal miRNA expression was assessed via NanoString Technology and microbiome signatures (MS) were assessed via shotgun metagenomics and non-negative matrix factorization. Ensemble machine learning classifiers were used to predict perMASLD using multi-omic and clinical features. Model performance was assessed using the area under the receiver operating characteristic curve (AUC).

**Results:**

Seven miRNAs were downregulated in patients with perMASLD (n = 14) and seven MS represented by bacteria genera *Blautia, Lachnospiraceae, Streptococcus, Faecalicabacterium, Akkermansia, Bifidobacterium,* and *Blautia/Anaerostipes* were associated with MO. Baseline alanine aminotransferase levels, impaired fasting glucose diagnosis at both baseline and 12-months post-RYGB and mir-4451 were the three most important predictors of perMASLD with model AUC= 84.5%.

**Conclusions:**

This study showed that specific fecal MS and miRNAs can predict perMASLD in patients with MO. Integrating multi-omic data with clinical features enabled robust prediction of perMASLD, highlighting the potential of non-invasive biomarkers to guide personalized interventions.

**Funding Agencies:**

CIHRBanting & Best Diabetes Centre, University of Toronto

